# Closing the gap: addressing telehealth disparities across specialties in the sustained pandemic era

**DOI:** 10.1038/s41746-024-01201-w

**Published:** 2024-08-21

**Authors:** Saki Amagai, Edward Vonesh, James Adams, Yuan Luo

**Affiliations:** 1 Department of Preventive Medicine, Northwestern University Feinberg School of Medicine, Chicago, IL USA; 2 Department of Emergency Medicine, Northwestern University Feinberg School of Medicine, Chicago, IL USA

**Keywords:** Health policy, Health services

## Abstract

Missed appointments, or no-shows, disrupt healthcare delivery, exacerbating chronic disease management and leading to worse health outcomes. Telehealth has surged as a viable solution to reduce no-shows and improve healthcare accessibility, especially during the COVID-19 pandemic. However, telehealth disparities and its long-term efficacy across various medical specialties remain understudied. To address this, we performed a retrospective analysis of electronic health records from a heterogenous network of hospitals in Illinois, examining telehealth use and no-shows across among 444,752 adult patients with 1,973,098 outpatient encounters across nine specialties during the sustained pandemic phase (i.e., January 1, 2021 to July 1, 2022). Among them, 84,290 (4.27%) were no-shows, and telehealth constituted 202,933 (10.3%) of the total encounters. Telehealth use during the sustained phase varied significantly by specialty type. Overall, telehealth encounters were associated with reduced no-show odds compared to in-person encounters (OR, 0.28; 95% CI, 0.26–0.29). Black and Hispanic patients, as well as those with Medicaid, had higher no-show odds relative to their counterparts, even when using telehealth. Mental health specialty had the highest telehealth usage rate and the highest no-show odds (OR, 2.99; 95% CI, 2.84–3.14) relative to other specialties included in the study. Moreover, specialty type had differential effects on no-shows for telehealth. These results underscore the variability in telehealth use by specialty type and pervasive disparities telehealth use and no-shows. As we move beyond the pandemic, our findings can inform policymakers to tailor policies and incentives to reach different patient groups as well as specialties, with varying needs, to promote equitable telehealth utilization.

## Introduction

Missed appointments (i.e., no-shows) can lead to fragmented care, inadequate management of chronic conditions, delayed diagnosis and treatment, and ultimately worse health outcomes^1,2^. Regular follow-up and continuity of care are crucial for effective treatment, preventative care, mental and psychosocial care, and chronic disease management^3,4^. No-shows could also prevent clinicians from reallocating those time slots or resources to other patients, resulting in inefficient healthcare delivery and loss of revenue for health systems^5^. Previous research has identified common reasons for no-shows, including access barriers such as cost and transportation, and other factors like forgetting appointments, work conflicts, childcare, and long wait times^6,7^. Patients of lower socioeconomic status are particularly vulnerable to missing medical appointments, as they encounter more pronounced barriers to access, especially regarding transportation and affordability^8,9^.

Telehealth, which saw a dramatic increase in use across U.S. health systems^10–13^ during the early phase of the COVID-19 pandemic, holds significant potential for reducing no-shows^14^ and, by extension, disparities in healthcare access. The greatest advantage to telehealth is its convenience. Unlike traditional in-person care, telehealth appointments require less time commitment, are more cost-effective, and can be easily accessed from anywhere at the patient’s convenience^15^, while minimizing exposure to communicable diseases, such as COVID-19. However, some earlier studies^16–21^ have identified disparities in telehealth access and use, particularly among racial/ethnic minorities, individuals with low socioeconomic status, and residents of rural areas.

Federal and state policy changes during COVID-19 have been instrumental in rapidly expanding telehealth. Medicare expanded coverage for the approved list of telehealth services beyond the Public Health Emergency through the end of 2023. In Illinois, Governor Pritzker signed a legislation signed in July 2021 that established permanent coverage for mental health via telehealth, while extending coverage for all other telehealth services until 2027^22^. Despite these policy changes to encourage telehealth use, its sustained use and its promise of reducing disparities remain poorly understood. Many previous studies^23–32^ on telehealth are narrowly focused either on a single specialty or on the early phase of the pandemic, thus questioning the appropriateness of applying these findings to understand implications of telehealth in other specialties nor determine directions to shape future policies beyond the pandemic.

While Bhatta et al^33^. and Chen et al^34^. did study telehealth no-shows during the later phase of the pandemic, their research was limited to behavioral health in rural areas and primary care in urban areas, respectively, which makes it challenging to generalize their findings more broadly. Sumarsono et al^28^. explored telehealth during the later phase and assessed no-shows across multiple specialties, yet they did not differentiate between video and telephone consultations. Prior studies have shown that video-consultations demonstrate greater clinical effectiveness, with higher diagnostic accuracy and decision-making accuracy, than audio-only calls^35^. Therefore, such an oversight is problematic, as the objective in medical consultations extends beyond minimizing no-shows but to also deliver high-quality care. Additionally, Sumarsono et al.’s inclusion of early pandemic data (i.e., March 2020) in the regression analyses—a period marked by rapid shifts in healthcare delivery—without adequately accounting for these dynamic changes, risks generating skewed interpretations. The constant evolution of the pandemic, characterized by emerging virus variants, new vaccinations, and evolving health policies, underscores the necessity for rigorous temporal controls in data analysis to ensure accurate interpretations.

To address these gaps, we performed a retrospective cohort analysis using electronic health records (EHR) data across nine adult specialties during the sustained phase, focusing on telehealth’s ongoing use and no-shows for video consultations. This broader approach helps understand telehealth’s effectiveness and informs future policies by highlighting areas needing targeted improvement to ensure equitable access.

## Results

### Population characteristics

Overall, we identified 444,752 unique patients and a total of 1,973,098 outpatient encounters (202,933 [10.28%] telehealth, 1,770,165 [89.72%] in-person) during the sustained pandemic phase (January 1, 2021–July 1, 2022). Of these, 84,280 (4.27%) were no-shows. Patient characteristics of all encounters during this sustained phase is shown in Table 1 (1,563,843 [79.26%] White, 211,455 [10.72%] Black, 197,800 [10.02%] Hispanic). Among these encounters, 1,264,702 (64.10%) were women and the mean (SD) age at first visit was of 54.29 (18.28) years.

**Table 1:** Study Sample Characteristics

	Black		White		Hispanic	
Characteristic	In-person (*n* = 186,927)	Telehealth (*n* = 24,528)	In-person (*n* = 1,405,374)	Telehealth (*n* = 158,469)	In-person (*n* = 177,864)	Telehealth (*n* = 19,936)
No-show Encounters (%)	21,009 (11.2)	809 (3.3)	47,094 (3.3)	2,433 (1.5)	12,464 (7.0)	471 (2.4)
**Sex, No. (%)**						
Female	132,555 (70.9)	19,516(79.6)	867,618 (61.7)	50,562 (68.1)	55,597 (68.7)	5,088 (74.5)
Male	54,372 (29.1)	5,012 (20.4)	537,756 (37.4)	50,562 (31.9)	55,597 (31.3)	5,088(25.5)
**Age at first visit, No. (%)**						
18–39	52,937 (28.3)	8,921 (30.1)	344,827 (24.5)	53,009 (33.5)	75,769 (42.6)	9,547 (47.9)
40–64	84.969 (45.5)	14,305(48.2)	561,885 (40.0)	66,111 (41.7)	72,628 (40.8)	8,373 (42.0)
65+	49,021 (26.2)	6,427 (21.7)	498,662 (35.5)	39,349 (24.8)	29,467 (16.6)	2,016 (10.1)
**Primary Payer, No. (%)**						
Commercial	81,721 (43.7)	11,682 (47.6)	772,402 (55.0)	101,291(63.9)	99,360 (55.9)	12,848 (64.4)
Medicaid	38,552 (20.6)	4,125 (16.8)	76,347 (5.4)	8,793 (5.5)	35,632 (20.0)	3,270 (16.4)
Medicare	65,351 (34.0)	8,375 (34.1)	534,323 (38.0)	45,914 (29.0)	35,055 (19.7)	3,329 (16.7)
Uninsured	3,103 (1.7)	346 (1.4)	22,302 (1.6)	2,471 (1.6)	7,817 (4.4)	489 (2.5)
**Median Household Income, No. (%)**						
<$50,000	84,655 (45.3)	10,810 (44.1)	75,300 (5.4)	7,837 (4.9)	27,286 (15.3)	3,058 (15.3)
$50,000–100,000	77,338 (41.4)	10,315 (42.1)	768,830 (54.7)	87.430 (55.2)	121,118 (68.1)	13,193 (66.2)
$100,000+	24,934 (13.3)	3,403 (13.9)	561,244 (39.9)	63,202 (39.9)	29,460 (16.6)	3,685 (18.5)
**Type of Visit, No. (%)**						
New Patient	34,367 (18.4)	2,888 (11.8)	237,167 (16.9)	19,488 (12.3)	35,566 (20.0)	2,671 (13.4)
Established Patient	54,372 (81.6)	5,012 (88.2)	537,756 (83.1)	50,562 (87.7)	55,597 (80.0)	5,088 (86.6)
**Charlson Comorbidity Index, No. (%)**						
0	56,148 (30.0)	7,122 (29.0)	743,313 (37.0)	62,294 (39.3)	76,162 (42.8)	8,057 (40.4)
1–2	55,398 (29.6)	8,013 (32.7)	649,477 (32.3)	54,678 (34.5)	55,688 (31.3)	6,957 (34.9)
3+	75,381 (40.3)	9,393 (38.3)	617,709 (30.7)	41,497 (26.2)	46,014 (25.9)	4,922 (24.7)
**Distance to Healthcare Facility, No. (%)**						
Q1 (0–3.1 miles)	27,066 (14.5)	3,503 (14.3)	386,433 (27.5)	43,862 (27.7)	27,673 (15.6)	3,376 (16.9)
Q2 (3.2–6.0 miles)	41,919 (22.4)	4,551 (18.6)	415,434 (29.6)	41,572 (26.2)	51,570 (29.0)	5,120 (25.7)
Q3 (6.1–12.2 miles)	70,208 (37.6)	9,210 (37.5)	328,263 (23.4)	34,674 (21.9)	65,638 (36.9)	7,116 (35.7)
Q4 (12.3 miles + )	47,733 (25.5)	7,264 (29.6)	275,243 (19.6)	38,361 (24.2)	32,983 (18.5)	4,324 (21.7)
**Specialty, No. (%)**						
Cardiology	22,947 (12.3)	470 (1.9)	134,883 (9.6)	3,985 (2.5)	14,256 (8.0)	259 (1.3)
Dermatology	11,978 (6.4)	221 (0.9)	133,124 (9.5)	1,733 (1.1)	12,578 (7.1)	285 (1.4)
Endocrinology	5,678 (3.0)	2,437 (9.9)	57,718 (4.1)	13,864 (8.7)	8,209 (4.6)	2,7107 (10.6)
Genetics	405 (0.2)	610 (2.5)	3,708 (0.3)	3,580 (2.3)	475 (0.3)	613 (3.1)
Mental Health	10,035 (5.4)	6,222 (25.4)	69,384 (4.9)	30,628 (19.3)	7,653 (4.3)	4,518 (22.7)
OBGYN	22,279 (11.9)	362 (1.5)	132,331 (9.4)	2,030 (1.3)	30,628 (17.2)	392 (2.0)
Oncology	23,209 (12.4)	1,076 (4.4)	180,283 (12.8)	9,503 (6.0)	18,734 (10.5)	789 (4.0)
Primary Care	85,114 (45.5)	12,706 (51.8)	656,198 (46.7)	90,855 (57.3)	81,367 (45.7)	10,758 (54.0)
Pulmonology	5,282 (2.8)	424 (1.7)	37,745 (2.7)	2,291 (1.4)	3,964 (2.2)	215 (1.1)
**Time, No. (%)**						
Jan–March, 2021	26,159 (14.0)	3,968 (16.2)	199,978 (14.2)	28,300 (17.9)	24,338 (13.7)	3,262 (16.4)
Apr–Jun, 2021	28,134 (15.1)	3,622 (14.8)	211,032 (15.0)	21,220 (13.4)	25,308 (14.2)	2,716 (13.6)
Jul–Sep, 2021	29,414 (15.7)	3,502 (14.3)	222,327 (15.8)	20,589 (13.0)	26,684 (15.0)	2,726 (13.7)
Oct–Dec, 2021	32,560 (17.4)	3,757 (15.3)	250,443 (17.8)	24,945 (15.7)	30,843 (17.3)	3,181 (16.0)
Jan–Mar, 2022	34,320 (18.4)	5,182 (21.1)	250,124 (17.8)	32,959 (20.8)	33,693 (18.9.)	4,293 (21.5)
Apr–Jun, 2022	36,340 (19.4)	4,497 (18.3)	271,470 (19.3)	30,456 (19.2)	36,998 (20.8)	3,758 (18.9)

Patient characteristics during the sustained pandemic phase (January 1, 2021–July 1, 2022) are described by encounter type and race/ethnicity categories.

### Temporal Trends from 2019 to 2022

**Fig. 1:**
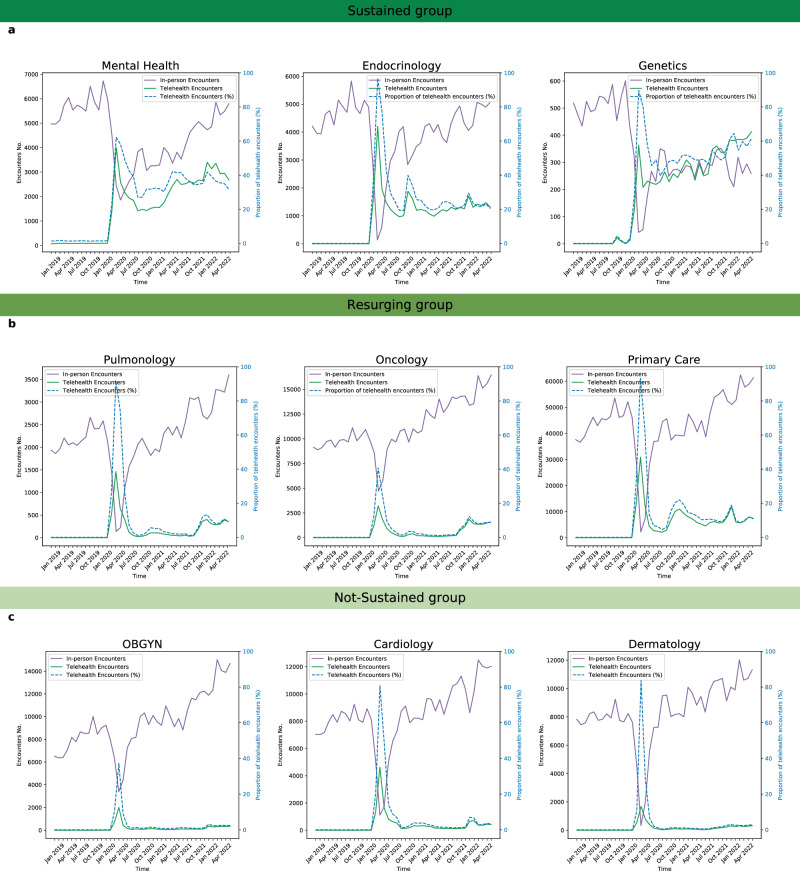
Telehealth usage across specialties before and during COVID-19. Only completed telehealth or in-person encounters were included. Telehealth usage was defined as the percentage of telehealth encounters (dashed line). This percentage calculated by dividing the number of completed telehealth encounters by the total number of completed encounters, where the total includes both in-person and telehealth encounters. **a** the Sustained group (mental health, endocrinology, and genetics) had higher telehealth usage even during the sustained phase **b** the Resurging group (primary care, pulmonology, and oncology) had increase usage over time **c** the Not-Sustained group (cardiology, dermatology, and OGBYN) remained low from May 2020 onward.

Prior to the COVID-19 pandemic, telehealth usage was virtually non-existent, rounding to 0% of outpatient encounters among all nine specialties (Fig. 1). At the onset of the pandemic when telehealth was rapidly adopted in March 2020, there is a notable spike in telehealth usage. During the sustained phase, telehealth usage varied among different specialties, both in terms of the proportion of total encounters and the duration of consistent usage. The Sustained group (mental health, endocrinology, and genetics) had higher telehealth usage even during the sustained phase, ranging from 19.8% to 64.5% of total completed encounters. While telehealth usage in the Not-Sustained group (cardiology, dermatology, and OBGYN) remained low from May 2020 onward, the usage in Resurging group (primary care, pulmonology, and oncology) has increased over time.

**Fig. 2:**
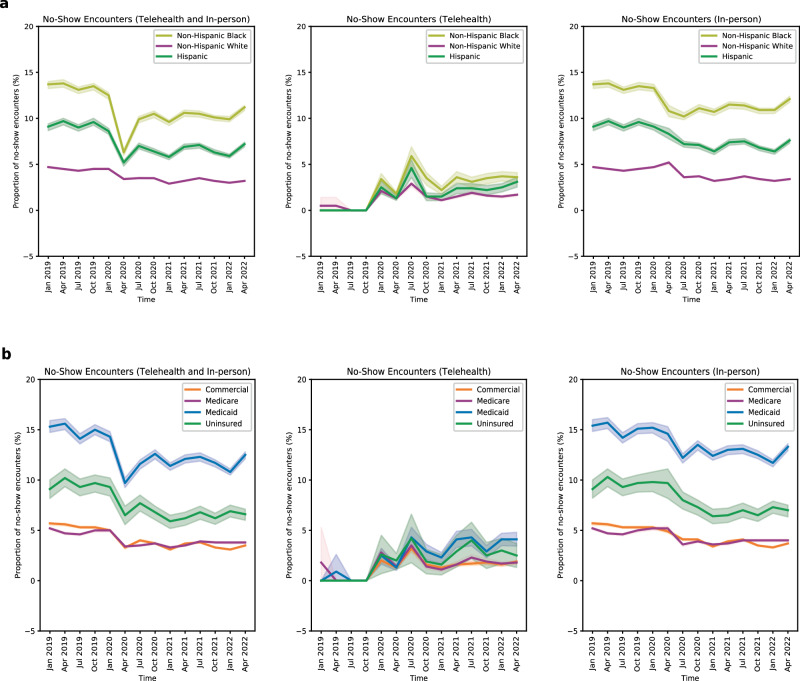
Temporal Trends in No-Show Rates. No-show rate, or percentage of no-show encounters, is determined by dividing the number of no-show encounters by the total number of scheduled encounters, where the total includes no-show, completed, and canceled encounters. All the nine specialties are aggregated in this temporal analysis. The 95% confidence interval bands represent the range within which we are 95% confident that the values for the observed trend data points lie. **a** Difference racial and ethnic groups exhibited varying no-show rates for in-person and telehealth appointments, yet telehealth seems to reduce these disparities across all specialties **b** No-show rates for in-person and telehealth appointments by insurance type, with Medicaid or uninsured patients having the highest rates, though these differences narrow with telehealth.

Disparate trends in no-show rates were observed among different racial/ethnic groups for in-person and telehealth encounters, yet telehealth appears to shrink this gap across all specialties (Fig. 2a). Prior to the emergence of the pandemic, the overall no-show rate for telehealth and in-person encounters were 13.5% (95% CI, 13.09–13.80%) for Black, 4.5% (95% CI, 4.44–4.60%) for White, and 9.6% (95% CI, 9.26–10.0%) for Hispanic patients in the last quarter of 2019. In response to the emergence of COVID-19, there was a temporary decline in no-show rates across all races as many healthcare appointments were not offered by providers. No-show rates have since increased, and disparities in no-show rates persisted during the study period. However, relative to pre-pandemic period, the overall no-show rates have decreased with increased telehealth usage in the sustained phase, such as in the second quarter of 2022 (11.2% [95%CI, 10.85–11.40%] Black, 3.2% [95% CI, 3.12%-3.20%] White, 7.2% [95% CI, 6.91–7.40%] Hispanic). When using telehealth, the differences in no-show rates between races appear to shrink (3.6% [95% CI, 3.03–4.10%] Black, 1.7% [95% CI, 1.53–1.80%] White, 3.1% [95%CI, 2.53–3.60%] Hispanic). Figure 2b presents no-show rates for in-person and telehealth encounters stratified by different types of insurance. Patients with Medicaid or no insurance had the highest no-show rates, though these differences appear to shrink with telehealth (Supplementary Information 1).

### Odds of no-show encounters

**Fig. 3:**
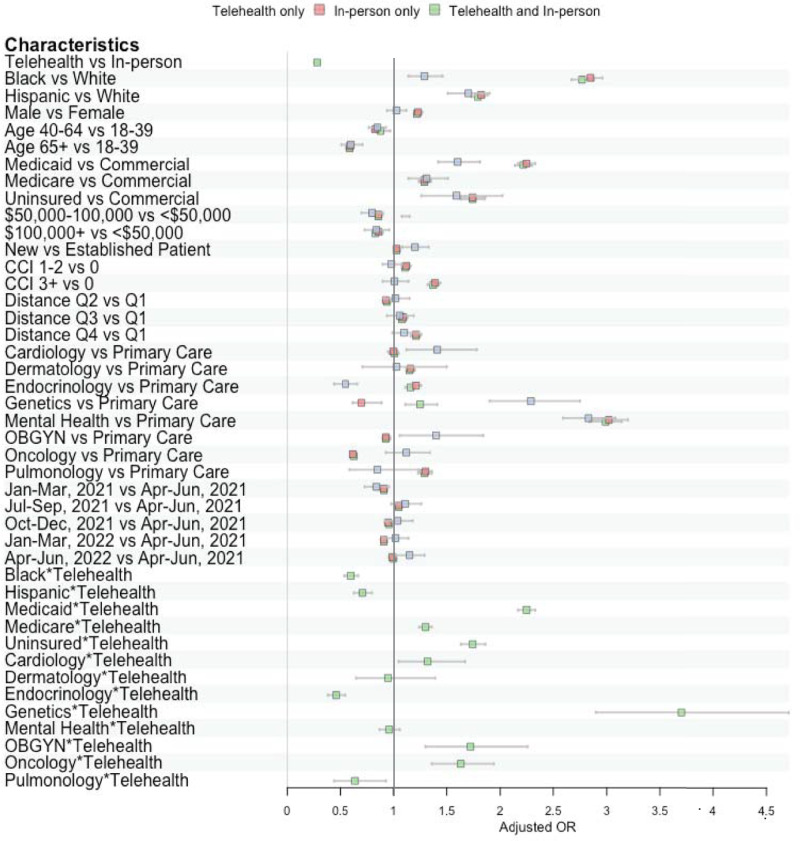
Forest plot of Adjusted Odds Ratio (OR) of No-Show. This forest plot displays the adjusted OR, calculated using a generalized estimation equation (GEE) multivariate logistic regression, which examines the no-show odd across different encounter modalities. The plot illustrates three distinct encounter categories: green for telehealth and in-person encounters; red for in-person only encounters; and blue for telehealth only encounters. Overall, telehealth was associated with reduced no-show odds compared to in-person encounters. OR odds ratio, CCI Charlson Comorbidity Index.

Overall, telehealth was associated with reduced no-show odds (adjusted odds ratio [aOR], 0.37; 95% CI, 0.35–0.40) compared to in-person encounters (Fig. 3**;** see Supplementary Table 1 for raw data). While racial disparities in no-show odds were observed, telehealth was associated with reducing these differences, with lower no-show odds for Hispanic and Black patients in telehealth appointments relative to White patients in in-person appointments (aOR=0.71, 95% CI = 0.63–0.80 and aOR = 0.60, 95% CI = 0.54-0.66, respectively). However, within telehealth, Black and Hispanic patients continued to have higher no-show odds than White patients (aOR = 1.29; 95%, 1.14–1.46 and aOR = 1.70; 95% CI = 1.51–1.90). Medicaid and Medicare patients using telehealth also showed lower no-show odds compared to those with commercial insurance attending in-person appointments (aOR=0.70, 95%CI = 0.61–0.79 and aOR=0.90, 95%CI = 0.82–0.99).

For mental health care, no-show odds were significantly higher (aOR=3.02; 95% CI = 2.85–3.19) compared to primary care, with telehealth not significantly associated with reducing these odds (aOR = 0.96, 95%CI = 0.87–1.06). Genetics showed significantly high no-show odds with telehealth relative to their reference group (aOR = 3.70, 95% CI = 2.91-4.71). Conversely, OBGYN, oncology, and cardiology similarly had higher no-show odds, while endocrinology and pulmonology saw reduced odds.

No-show odds for in-person encounters increased significantly with greater distance to the clinic, more comorbidities, and being male; however, these factors were not statistically significant for telehealth encounters. Younger patients had higher no-show odds for both telehealth and in-person encounters. While no-show odds for in-person encounters varied significantly over time, those for telehealth remained more stable.

### Stratified analyses on race/ethnicity and insurance type

**Fig. 4:**
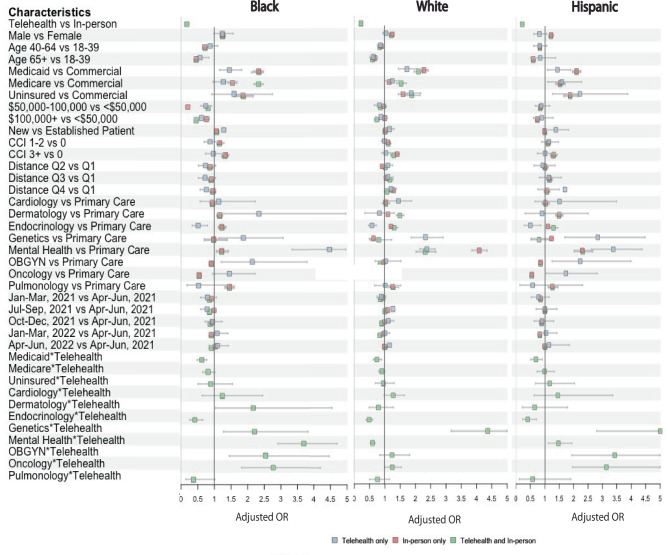
Forest plot of Adjusted Odds of No-Show Encounters by Race/Ethnicity and Encounter Type. This forest plot displays the adjusted OR, calculated using generalized estimation equation (GEE) multivariate logistic regression stratified by race/ethnicity. The outcome is the no-show odd across different encounter modalities. The plot illustrates three distinct encounter categories: green for telehealth and in-person encounters; red for in-person only encounters; and blue for telehealth only encounters. OR odds ratio, CCI Charlson Comorbidity Index.

Stratifying by race/ethnicity and encounter type in Fig. 4 (see Supplementary Table 2-4 for detailed data) revealed differential no-show odds within mental health care compared to primary care. Telehealth was associated with significantly higher no-show odds for Black and Hispanic patients seeking mental health services (aOR=3.69; 95% CI = 2.91–4.69 and aOR=1.47; 95% CI = 1.12–1.94), while White patients experienced lower no-show odds with telehealth (aOR = 0.6; 95% CI = 0.53–0.68). In-person care showed higher no-show odds for patients with more comorbidities across all racial/ethnic groups, a trend not significantly present in telehealth encounters.

Moreover, for all race/ethnicity groups, telehealth was associated with reduced no-show odds among Medicaid patients compared to their respective in-person encounters. Even when using telehealth, Medicaid patients showed higher no-show odds relative to those with private insurance across these groups, suggesting ongoing access barriers for low socioeconomic groups. Stratified analysis by insurance type showed similar overall trends (Supplementary Table 5).

### Mental health care

In our sub-analysis on mental health care (Supplementary Information 1 and Supplementary Table 6), we also observed racial/ethnic and insurance disparities (Supplementary Fig. 1). Interestingly, older mental health patients (65+) were significantly more likely to miss appointments (aOR = 1.83, 95%CI = 1.58–2.12), deviating from the general trend of higher no-show rates among younger patients (Supplementary Table 6 and Supplementary Information 2).

## Discussion

Our findings shed light on telehealth disparities and no-shows across diverse medical specialties, race/ethnicity groups, and insurance types during the *sustained* phase of the pandemic. In light of existing literature, this study stands as one of the first to delve into the long-term telehealth usage patterns for video-based medical consultations and their implications across multiple medical fields. Previous research typically examined telehealth usage and no-show patterns during the early stages of the pandemic or in isolated medical specialties^16,18–21,36^. We have observed that while telehealth has significantly reduced no-shows, disparities continue to exist. Telehealth has been continuously used beyond the onset of the pandemic, particularly within the Sustained group, including mental health, endocrinology, and genetics. This persistent usage suggests telehealth’s potential for permanent integration into standard healthcare practices. An interesting surge in telehealth usage was observed from January 2022 onward in the Resurging group (i.e., primary care, pulmonology, and oncology), reflecting healthcare’s dynamic adaptations to the evolving pandemic. This increase in telehealth use for the Resurging group could be due to primary care and pulmonology’s active involvement in COVID-19 treatment, necessitating social distancing during case surges. Oncology patients, like those in pulmonology, often have chronic conditions that increase vulnerability to severe illnesses, may also benefit from the reduced exposure that telehealth provides.

Despite these positive trends, the anticipated levelling of the healthcare playing field through telehealth has not been fully realized, as indicated by persisting disparities. Previous studies have shown that telehealth reduces no-shows^14,23,25,37^ and, thereby, deeming it a success; however, it is equally important to explore the ongoing disparities within telehealth, which highlight persistent challenges that need to be assessed for more improvement. Specifically, Black and Hispanic patients continued to experience higher no-show odds relative to White patients even when using telehealth. In our race-stratified analysis, the higher no-show odds among Medicaid patients across all races suggest that, while telehealth has generally reduced missed appointments, it has not fully overcome the longstanding access barriers faced by individuals of lower socioeconomic status. These barriers may include technological limitations^35^, conflicting work schedules, transportation issues^38^, and other pressing life responsibilities^39,40^ that are often more burdensome for low-income populations. It is, therefore, still too premature to assert that disparities issues are eliminated with telehealth use, as suggested in previous studies^41,42^.

Broader factors like comorbidities and distance to clinic played a significant role in no-shows across all specialties. Patients with multiple health issues are less likely to attend in-person visits, yet telehealth’s convenience may alleviate this issue, emphasizing its utility for those with complex health needs of living far from healthcare facilities. Moreover, the lesser impact of distance on telehealth attendance highlights its effectiveness in bridging geographical gaps, suggesting that telehealth can enhance healthcare access, particularly for remote populations.

Our findings also highlight challenges specific to mental health services. Despite the highest utilization of telehealth, mental health had the highest no-show odds relative to other specialties, regardless of modality type. This may be because patients in other specialties have more urgent needs for their appointments, whereas mental health patients may face conditions like anxiety or depression that may affect motivation and ability to keep appointments. Additionally, telehealth was not associated with reduced no-show odds for mental health care, underscoring the complexity of patient engagement in this specialty. Factors such as patient’s discomfort for sensitive discussions and challenges in developing patient-clinician relationship via telehealth may contribute to this issue^43^. When stratifying by race/ethnicity, Black and Hispanic patients seeking mental healthcare had higher no-show odds for telehealth than their counterparts while the opposite trend was true for White patients, suggesting a potential “digital divide”^44,45^.

Beyond mental health, telehealth did not uniformly reduce no-shows across all specialties, revealing nuanced challenges in its implementation. While endocrinology and pulmonology experienced substantial declines in no-show odds with, others such as, genetics OBGYN, oncology, and cardiology saw increases. These differences suggest that the no-shows for telehealth are also contingent on the specific nature of the medical practice and the distinct needs of the patient population served.

Our findings have important implications for shaping future telehealth policies. First, the apparent racial and ethnic disparities coupled with economic disadvantage that are jeopardizing access to care, require national endeavors aimed at eradicating these discrepancies. These efforts must encompass not only reforms within the health care system but also policy interventions that address nonmedical barriers (e.g., transportation, employment, education, and technology) to accessing and receiving quality healthcare^46,47^.

Second, due to the high variability in telehealth use across specialties over time, tailored efforts to enhance use are warranted. For examples, specialties less inclined to use telehealth due to reliance on physical examinations (i.e., Not-Sustained group) may benefit from efforts to integrate telehealth for follow-up^48^. This may be especially true for going over lab/test results, confirming medicine regimen, or monitoring chronic conditions with routine longitudinal care, yet more efforts may be needed to build overall trust in virtual care among physicians and patients alike (Supplementary Information 3). Telehealth could mitigate long waits for the patients with faster and safer consultations. Physicians could also benefit from more flexible, remote working arrangements facilitated by the pandemic’s push for virtual consultations.

This study has limitations. First, our study focused on a network of hospitals in one state, which may limit the generalizability of the findings to other healthcare systems or states with different demographics and policies. Second, our study relied on self-identified race and ethnicity data from patient registration, which may be subject to misclassification. Third, our study utilized a retrospective cohort design, which can only establish associations rather than causation.

In summary, our study contributes to an understanding of telehealth’s evolving role and the entrenched disparities in its use and no-shows during the sustained phase of the pandemic. The findings provide a foundation for future research to inform nuanced policy decisions that promote equitable healthcare access across different patient groups and medical specialties as we move beyond the pandemic.

## Methods

### Study setting and data collection

In this retrospective cohort study, we conducted an EHR review using outpatient care data from a network of hospitals in Illinois, which includes 11 hospitals and over 200 clinics, to estimate changes in telehealth usage across different demographic groups. This study was approved as minimal risk and exempt from the requirement for informed consent by Northwestern University Institutional Review Board due to its sole use of de-identified retrospective information. We followed the Strengthening the Reporting of Observational Studies in Epidemiology (STROBE) reporting guideline.

Our unit of analysis was a patient encounter for outpatient care services. An encounter was defined as a patient seeing a specific practitioner at a specific time in a specific health facility. We considered telehealth service as virtual care provided on a video telehealth platform, as video-consultations have demonstrated enhanced clinical efficacy than audio-only calls. Completion of either telehealth or in-person encounter was defined as a completion of the encounter, as indicated by the EHR indicator (in contrast to being canceled or resulting in a no-show). Canceled encounters were canceled by either the patient or their provider, irrespective of whether the appointment was later rescheduled. No-show encounters indicate that the patient failed to attend the scheduled appointment.

### Study participants and patient characteristics

Using EHR data, we extracted demographic information for adult patients (aged $\ge \,$18) residing in Illinois and scheduled in our health system’s care and medical specialty clinics between January 1, 2019 and July 1, 2022. Race and ethnicity were self-identified. Patient-selected primary race and ethnicity, without overlap, were consolidated into the following aggregated categories: Hispanic, Non-Hispanic Black, and Non-Hispanic White. We focused on Hispanic and Black patients because these demographic groups have historically faced disparities in accessing U.S. healthcare. Charlson Comorbidity Index (CCI) was used as a proxy for chronic conditions, and the severity of comorbidity was categorized into three grades as described in a prior study^49^: mild, with CCI scores of 1–2; moderate, with CCI scores of 3–4; and severe with CCI scores greater than 5. The nine medical specialties selected for analysis were primary care and specialty care (cardiology, dermatology, endocrinology, genetics, pulmonology, obstetrics and gynecology, and oncology, and mental health). These specialties were purposively selected to capture potential variation in telehealth usage, based on an analysis conducted among adult specialties at the onset of pandemic^50^.

We used the patients’ primary insurance type and the median per capita income of residential zip code obtained from the American Community Survey as proxies for socioeconomic status. A new patient was defined as a patient who had not received any professional services from the same physician group specialty within the previous 3 years^51^. Distance for each patient to access care was calculated using geopy version 2.3.0^52^. The centroid of each patient’s self-reported zip code at baseline and provider addresses were converted to identifiable coordinates using Google Maps API^53^.

### Statistical analysis

We first described the general trends of telehealth usage and no-shows. Telehealth usage was defined as the total number of completed telehealth encounters divided by the total number of any completed encounter, including both in-person and telehealth encounters. Medical specialties were categorized into three groups according to their telehealth use during the pandemic’s sustained phase: sustained group (20%), resurging group (5–20% with a notable increase from January 2022 onward), and non-sustained group ( < 5%). No-show rate was defined as the total number of no-show encounters divided by the total number of scheduled encounters.

To assess the association between patient characteristics (e.g., race/ethnicity) and no-show encounters, we used generalized estimation equation (GEE) multivariate logistic regression which takes account for the within-patient correlation between repeated patient encounters. All analyses utilize robust standard errors and a working independence correlation structure for repeated binary outcomes. Resulting fixed-effects estimates were exponentiated to generate adjusted odds ratio. For all analyses, a 2-sided *P* < 0.05 was used to determine statistical significance. All results are reported with 95% CIs. We focused our analyses on the sustained pandemic phase and included the encounters between January 1, 2021 (when COVID-19 vaccines started to become available to the general population in Illinois) and July 1, 2022. The deployment of COVID-19 vaccines represented a watershed moment in managing the pandemic (by reducing disease severity, transmission rates, and aiding in economic and social recovery), leading Illinois into a new phase. Due to the inability to accurately identify the reasons for cancellations, we chose to exclude canceled appointments from our analyses. The frequency of missing data was low ( < 4%) across all variables and therefore missing data were excluded from the analysis. All statistical analyses were conducted using Python version 3.9.12.

To further explore differences in no-show, we evaluated whether there are any effect modifications (i.e., interactions) associated with encounter type (telehealth versus in-person encounters) according to race/ethnicity, insurance type, and medical specialties. Additionally, we conducted stratified analyses by encounter type, race/ethnicity, insurance type, and specifically within the subset of mental health care, rather than across all medical specialties.

### Supplementary information

Supplementary material

## Data Availability

The datasets used in this study are not openly accessible. This is due to valid privacy and security considerations, as electronic health records (EHR) data cannot be freely redistributed to researchers who are not part of the Institutional Review Board-sanctioned research collaborations with the named medical centers. Details regarding the data and how they can be accessed for legitimate research purposes can be provided upon reasonable request.

## Code availability

All the code used to prepare the dataset, run statistical models, and evaluate the conclusions of this work can be found in our GitHub repository (https://github.com/amagais/telehealth).
