# Supplementary material for: Closing the gap: addressing telehealth disparities across specialties in the sustained pandemic era
Source: NPJ Digit Med. 2024 Aug 21;7:217. doi: 10.1038/s41746-024-01201-w (PMC11335954; doi:10.1038/s41746-024-01201-w)
Supplement: Supplementary file 1 — Supplementary material [file 41746_2024_1201_MOESM1_ESM.pdf]

Supplemental Materials

SupplementaryTable1. Adjusted Odds of No-Show by Encounter Type

	Telehealth only		In-person only		Telehealth and In-person	
**Characteristics**	OR (95% CI)	P-value	OR (95% CI)	P-value	OR (95% CI)	P-value
**Encounter Type**						
In-person	NA	NA	NA	NA	1[Reference]	NA
Telehealth	NA	NA	NA	NA	0.28 (0.26-0.29)	<.001
**Race/Ethnicity**						
White	1[Reference]	NA	1[Reference]	NA	1[Reference]	NA
Black	1.29 (1.14-1.46)	<.001	2.85 (2.74-2.96)	<.001	2.77 (2.67-2.88)	<.001
Hispanic	1.70 (1.51-1.90)	<.001	1.82 (1.76-1.88)	<.001	1.79 (1.73-1.85)	<.001
**Sex**						
Male[a]	1.03 (0.94-1.12)	.544	1.23 (1.20-1.27)	<.001	1.22 (1.19-1.26)	<.001
**Age at First Visit**						
18-39	1[Reference]	NA	1[Reference]	NA	1[Reference]	NA
40-64	0.85 (0.77-0.93)	.001	0.83 (0.80-0.86)	<.001	0.88 (0.80-0.97)	.010
65+	0.60 (0.51-0.71)	<.001	0.59 (0.56-0.62)	<.001	0.59 (0.56-0.62)	<.001
**Insurance**						
Commercial	1[Reference]	NA	1[Reference]	NA	1[Reference]	NA
Medicaid	1.60 (1.42-1.81)	<.001	2.25 (2.17-2.33)	<.001	2.22 (2.14-2.30)	<.001
Medicare	1.31 (1.14-1.51)	<.001	1.29 (1.24-1.35)	<.001	1.29 (1.24-1.35)	<.001
Uninsured	1.59 (1.26-2.02)	<.001	1.74 (1.63-1.86)	<.001	1.74 (1.63-1.85)	<.001
**Median Household Income**						
<$50,000	1[Reference]	NA	1[Reference]	NA	1[Reference]	NA
$50,000-100000	0.80 (0.70-0.90)	<.001	0.86 (0.83-0.90)	<.001	0.86 (0.83-0.89)	<.001
$100000+	0.84 (0.73-0.96)	.011	0.86 (0.82-0.90)	<.001	0.86 (0.82-0.90)	<.001
**Type of Visit**						
New Patient[b]	1.20 (1.08-1.33)	<.001	1.03 (1.01-1.05)	.01	1.03 (1.01-1.06)	.002
**Charlson Comorbidity Index**						
0	1[Reference]	NA	1[Reference]	NA	1[Reference]	NA
1-2	0.98 (0.90-1.08)	.74	1.12 (1.08-1.16)	<.001	1.11(1.08 - 1.15)	<.001
3+	1.01 (0.90-1.14)	.82	1.39 (1.34-1.44)	<.001	1.37 (1.32-1.42)	<.001
**Distance to Healthcare Facility**						
Q1 (0-3.1 miles)	1[Reference]	NA	1[Reference]	NA	1[Reference]	NA
Q2 (3.2-6.0 miles)	1.02 (0.91-1.15)	.73	0.93 (0.90-0.97)	<.001	1.94 (0.91-0.97)	<.001
Q3 (6.1-12.2 miles)	1.06 (0.94-1.19)	.33	1.09 (1.05-1.13)	<.001	1.08 (1.05-1.12)	<.001
Q4 (12.3 miles+)	1.10 (0.99-1.24)	.080	1.21 (1.17-1.26)	<.001	1.21 (1.16-1.25)	<.001
**Specialty**						
Primary Care	1[Reference]	NA	1[Reference]	NA	1[Reference]	NA

Cardiology	1.41 (1.12-1.78)	.004	1.00 (0.95-1.04)	.90	1.01 (0.96-1.05)	.82
Dermatology	1.03 (0.71-1.50)	.88	1.16 (1.12-1.19)	<.001	1.15 (1.12-1.20)	<.001
Endocrinology	0.55 (0.46-0.66)	< .001	1.21 (1.16-1.26)	<.001	1.16 (1.11-1.20)	<.001
Genetics	2.29 (1.90-2.75)	< .001	0.74 (0.62-0.89)	.001	1.25 (1.11-1.41)	<.001
Mental Health	2.83 (2.59-3.08)	< .001	3.02 (2.85-3.20)	<.001	2.99 (2.84-3.14)	<.001
OBGYN	1.40 (1.06-1.84)	.01	0.93 (0.90-0.97)	<.001	0.93 (0.90-0.97)	<.001
Oncology	1.12 (0.93-1.34)	.23	0.62 (0.60-0.64)	<.001	0.63 (0.61-0.65)	<.001
Pulmonology	0.85 (0.59-1.24)	.40	1.30 (1.24-1.36)	<.001	1.29 (1.23-1.36)	<.001
**Time**						
January -March, 2021	0.84 (0.73-0.96)	.01	0.91 (0.89-0.94)	<.001	0.91 (0.88-0.94)	<.001
April – June, 2021	1[Reference]	NA	1[Reference]	NA	1[Reference]	NA
July – September, 2021	1.11 (0.98-1.26)	.10	1.05 (1.02-1.08)	.001	1.05 (1.02-1.08)	.001
October – December, 2021	1.04 (0.92-1.18)	.50	0.95 (0.92-0.98)	.002	0.96 (0.93-0.99)	.004
January – March, 2022	1.02 (0.90-1.14)	.79	0.91 (0.88-0.93)	<.001	0.91(0.89-0.94)	<.001
April – June, 2022	1.15 (1.02-1.29)	.02	0.99 (0.96-1.02)	.65	1.00 (0.97-1.03)	.92
**Interactions**						
Black*Telehealth	NA	NA	NA	NA	0.71 (0.63-0.81)	<.001
Hispanic*Telehealth	NA	NA	NA	NA	0.60 (0.54-0.66)	<.055
Medicaid*Telehealth	NA	NA	NA	NA	0.70 (0.61-0.79)	<.001
Medicare*Telehealth	NA	NA	NA	NA	0.90 (0.82-0.99)	.03
Uninsured*Telehealth	NA	NA	NA	NA	0.96 (0.75-1.22)	.69
Cardiology*Telehealth	NA	NA	NA	NA	1.32 (1.04-1.67)	.02
Dermatology*Telehealth	NA	NA	NA	NA	0.95 (0.65-1.39)	.78
Endocrinology*Telehealth	NA	NA	NA	NA	0.46 (0.39-0.55)	<.001
Genetics*Telehealth	NA	NA	NA	NA	3.7 (2.9-4.7)	<.001
Mental Health*Telehealth	NA	NA	NA	NA	0.96 (0.87-1.06)	.051
OBYGN*Telehealth	NA	NA	NA	NA	1.72 (1.34-2.27)	<.001
Oncology*Telehealth	NA	NA	NA	NA	1.63 (1.36-1.94)	<.001
Pulmonology*Telehealth	NA	NA	NA	NA	0.64 (0.44-0.93)	.018

Abbreviations: NA, not applicable; OR, odds ratio

[a] Reference group is female

[b] Reference group is Established Patient

Supplementary Table2. Stratified Analysis (Black Patients): Adjusted Odds of No-Show Encounters by Encounter Type

	Black					
**Characteristics**	Telehealth		In-person		Telehealth and In-person	
**Encounter Type**	OR (95% CI)	P-value	OR (95% CI)	P-value	OR (95% CI)	P-value
**In-person**	NA	NA	NA	NA	1[Reference]	NA
**Telehealth**	NA	NA	NA	NA	0.27 (0.14-0.20)	<.001
**Sex**						
Male[a]	1.24 (0.99-1.56)	.06	1.25 (1.18-1.32)	<.001	1.24 (1.17-1.31)	<.001
**Age at first visit**						
18-39	1[Reference]	NA	1[Reference]	NA	1[Reference]	NA
40-64	0.87 (0.69-1.10)	.25	0.71 (0.66-0.76)	<.001	0.71 (0.67-0.76)	<.001
65+	0.57 (0.40-0.83)	<.001	0.45 (0.40-0.49)	<.001	0.45 (0.41-0.50)	<.001
**Insurance**						
Commercial	1[Reference]	NA	1[Reference]	NA	1[Reference]	NA
Medicaid	1.45 (1.16-1.82)	.001	2.33 (2.18-2.47)	<.001	2.32 (2.19-2.47)	<.001
Medicare	1.27 (0.96-1.68)	.09	1.54 (1.41-1.67)	<.001	1.53 (1.41-1.67)	<.001
Uninsured	1.59 (0.92-2.74)	.09	1.86 (1.60-2.17)	<.001	1.86 (1.60-2.16)	<.001
**Median Household Income**						
<$50,000	1[Reference]	NA	1[Reference]	NA	1[Reference]	NA
$50,000-100,000	0.74 (0.59-0.91)	.004	0.2 (0.77-0.86)	<.001	0.82 (0.77-0.86)	<.001
$100,000+	0.61 (0.44-0.84)	.003	0.76 (0.70-0.84)	<.001	0.76 (0.70-0.83)	<.001
**Type of visit**						
New Patient[b]	1.28 (1.02-1.61)	.03	1.06 (1.02-1.11)	.008	1.07 (1.03-1.12)	.004
**Charlson Comorbidity Index**						
0	1[Reference]	NA	1[Reference]	NA	1[Reference]	NA
1-2	0.87 (0.69-1.10)	.24	1.16 (1.10-1.29)	<.001	1.15 (1.08-1.23)	<.001
3+	0.97 (0.73-1.28)	.82	1.33 (1.24-1.43)	<.001	1.32 (1.23-1.41)	<.001
**Distance to Healthcare Facility**						
Q1 (0-3.1 miles)	1[Reference]	NA	1[Reference]	NA	1[Reference]	NA
Q2 (3.2-6.0miles)	0.73 (0.52-1.04)	.09	0.87 (0.79-0.96)	.003	0.86 (0.78-0.95)	.002
Q3(6.1-12.2 miles)	0.72 (0.52-1.04)	.05	0.91 (0.83-0.99)	.04	0.91 (0.82-0.99)	.024
Q4 (12.3 miles+)	0.76 (0.57-1.03)	.07	0.96 (0.88-1.04)	.32	0.95 (0.87-1.04)	.24
**Specialty**						
Primary Care	1[Reference]	NA	1[Reference]	NA	1[Reference]	NA
Cardiology	1.13 (0.57-2.23)	.73	0.94 (0.86-1.03)	.15	0.94 (0.86-1.04)	.15
Dermatology	2.34 (1.11-4.96)	.03	1.16 (1.08-1.25)	<.001	1.17 (1.08-1.25)	<.001
Endocrinology	0.51 (0.33-0.79)	.003	1.22 (1.12-1.34)	<.001	1.23 (1.12-1.34)	<.001
Genetics	1.87 (1.14-3.07)	.013	0.98 (0.70-1.38)	.90	0.99 (0.70-1.34)	.91
Mental Health	4.47 (3.34-4.96)	<.001	1.22 (1.06-1.42)	.007	1.22 (1.06-1.41)	.007
OBGYN	2.14 (1.21-3.78)	.009	0.91 (0.85-0.97)	.008	0.91 (0.85-0.97)	.008
Oncology	1.45 (0.95-2.22)	.08	0.54 (0.49-0.58)	<.001	0.54 (0.50 -0.58)	<.001
Pulmonology	0.52 (0.18-1.42)	.20	1.45 (1.32-1.59)	<.001	1.45 (1.32-1.59)	<.001

**Time**						
Jan – Mar, 2021	0.79(0.58-1.06)	.12	0.91 (0.85-0.96)	.001	0.89 (0.84-0.95)	.001
Apr – Jun, 2021	1[Reference]	NA	1[Reference]	NA	1[Reference]	NA
Jul – Sep, 2021	0.78(0.59-1.05)	.10	0.99 (0.94-1.05)	.73	0.98 (0.93-1.04)	.52
Oct – Dec, 2021	0.94 (0.72-1.23)	.66	0.93 (0.87-0.98)	.009	0.92 (0.87-0.98)	.008
Jan – Mar, 2022	1.08 (0.84-1.40)	.55	0.91 (0.86-0.96)	.001	0.91 (0.86-0.96)	.002
Apr – Jun, 2022	1.09 (0.84-1.41)	.52	1.02 (0.96-1.09)	.001	1.02 (0.96-1.09)	.45
**Interactions**						
Medicaid*Telehealth	NA	NA	NA	NA	0.61 (0.48-0.77)	<.001
Medicare*Telehealth	NA	NA	NA	NA	0.81 (0.65-1.01)	.06
Uninsured*Telehealth	NA	NA	NA	NA	0.89 (0.51-1.54)	.67
Cardiology*Telehealth	NA	NA	NA	NA	1.24 (0.62-2.45)	.54
Dermatology*Telehealth	NA	NA	NA	NA	2.17 (1.04-4.55)	.04
Endocrinology*Telehealth	NA	NA	NA	NA	0.41 (0.26-0.64)	<.001
Genetics*Telehealth	NA	NA	NA	NA	2.21 (1.28-3.81)	.005
Mental Health*Telehealth	NA	NA	NA	NA	3.69 (2.91-4.69)	.12
OBYGN*Telehealth	NA	NA	NA	NA	2.54 (1.45-4.46)	.001
Oncology*Telehealth	NA	NA	NA	NA	2.76 (1.82-4.19)	<.001
Pulmonology*Telehealth	NA	NA	NA	NA	0.37 (0.14-1.01)	.05

Supplementary Table3. Stratified Analysis (White Patients): Adjusted Odds of No-Show Encounters by Encounter Type

	White					
**Characteristics**	Telehealth		In-person		Telehealth and In-person	
**Encounter Type**	OR (95% CI)	P-value	OR (95% CI)	P-value	OR (95% CI)	P-value
In-person	NA	NA	NA	NA	1[Reference]	NA
Telehealth	NA	NA	NA	NA	0.42 (0.39-0.45)	<.001
**Sex**						
Male[a]	1.03 (0.93-1.13)	.69	1.24 (1.20-1.28)	<.001	1.23 (1.19-1.27)	<.001
**Age at first visit**						
18-39	1[Reference]	NA	1[Reference]	NA	1[Reference]	NA
40-64	0.85 (0.76 -0.95)	.003	0.89 (0.85-0.93)	<.001	0.88 (0.85-0.92)	<.001
65+	0.62 (0.51-0.76)	<.001	0.67 (0.64-0.73)	<.001	0.69 (0.64-0.73)	<.001
**Insurance**						
Commercial	1[Reference]	NA	1[Reference]	NA	1[Reference]	NA
Medicaid	1.72 (1.44-2.05)	<.001	2.28 (2.15-2.42)	<.001	2.28 (2.19-2.42)	<.001
Medicare	1.24 (1.03-1.47)	.02	1.14 (1.08-1.21)	<.001	1.16 (1.08-1.22)	<.001
Uninsured	1.45 (1.08-1.96)	.01	1.59 (1.44-1.74)	<.001	1.58 (1.44-1.73)	<.001
**Median Household Income**						
<$50,000	1[Reference]	NA	1[Reference]	NA	1[Reference]	NA
$50,000-100,000	0.82 (0.66-1.00)	.06	0.96 (0.89-1.05)	.38	0.96 (0.88-1.04)	.29
$100,000+	0.87 (0.71-1.08)	.21	0.99 (0.91-1.07)	.72	0.98 (0.91-1.07)	.62
**Type of visit**						
New Patient[b]	1.15 (1.02-1.30)	.03	1.02 (0.99-1.04)	.29	1.02 (0.99-1.04)	.16
**Charlson Comorbidity Index**						
0	1[Reference]	NA	1[Reference]	NA	1[Reference]	NA
1-2	0.98 (0.89-1.10)	.74	1.09 (1.05-1.13)	<.001	1.09 (1.04-1.13)	<.001
3+	1.03(0.90-1.18)	.62	1.40 (1.34-1.47)	<.001	1.38 (1.32-1.45)	<.001
**Distance to Healthcare Facility**						
Q1 (0-3.1 miles)	1[Reference]	NA	1[Reference]	NA	1[Reference]	NA
Q2 (3.2-6.0miles)	1.10 (0.96-1.25)	.17	0.91 (0.87-0.95)	<.001	0.92 (0.88-0.95)	<.001
Q3(6.1-12.2 miles)	1.10 (0.96-1.27)	.17	1.04 (1.00-1.09)	.04	1.05 (1.01-1.09)	.02
Q4 (12.3 miles+)	1.20 (1.05-1.37)	.006	1.28 (1.23-1.34)	<.001	1.28 (1.23-1.33)	<.001
**Specialty**						
Primary Care	1[Reference]	NA	1[Reference]	NA	1[Reference]	NA
Cardiology	1.44 (1.11-1.87)	.006	1.02 (0.96-1.08)	.57	1.02 (0.97-1.08)	.45
Dermatology	0.82 (0.35-1.31)	.41	1.10 (1.06-1.14)	<.001	1.10 (1.06-1.14)	<.001
Endocrinology	0.58 (0.47-0.72)	<.001	1.20 (1.14-1.27)	<.001	1.21 (1.13-1.27)	<.001
Genetics	2.33 (1.86-2.91)	<.001	0.63 (0.49-0.81)	<.001	0.63 (0.49-0.81)	<.001
Mental Health	2.37 (2.14-2.64)	<.001	4.09 (3.85-4.35)	<.001	4.08 (3.83-4.32)	<.001
OBGYN	1.03 (0.69-1.52)	.89	0.96 (0.92-1.01)	.09	0.95 (0.91-0.99)	.04
Oncology	0.97 (0.78-1.21)	.79	0.56 (0.64-.0.71)	<.001	0.68 (0.65 -0.71)	<.001
Pulmonology	1.01 (0.67-1.52)	.98	1.25 (1.18-1.33)	<.001	1.25 (1.18-1.33)	<.001

**Time**						
Jan – Mar, 2021	0.88 (0.74-1.03)	.11	0.94 (0.90-0.97))	.001	0.93 (0.89-0.97)	<.001
Apr – Jun, 2021	1[Reference]	NA	1[Reference]	NA	1[Reference]	NA
Jul – Sep, 2021	1.26 (1.09-1.16)	.002	1.09 (1.04-1.13)	<.001	1.09 (1.05-1.13)	<.001
Oct – Dec, 2021	1.11 (0.95-1.29)	.186	0.98 (0.94-1.13)	.34	0.99 (0.95-1.03)	.49
Jan – Mar, 2022	1.00 (0.86-1.16)	.99	0.94(0.90-0.97)	<.001	0.94 (0.90-0.97)	.001
Apr – Jun, 2022	1.15 (0.99-1.13)	.06	0.98 (0.94-1.02)	.28	0.99 (0.95-1.02)	.48
**Interactions**						
Medicaid*Telehealth	NA	NA	NA	NA	0.74 (0.62-0.88)	.001
Medicare*Telehealth	NA	NA	NA	NA	0.90 (0.80-1.00)	.06
Uninsured*Telehealth	NA	NA	NA	NA	0.95 (0.70-1.30)	.78
Cardiology*Telehealth	NA	NA	NA	NA	1.27 (0.98-1.64)	.80
Dermatology*Telehealth	NA	NA	NA	NA	0.79 (0.39-1.27)	.34
Endocrinology*Telehealth	NA	NA	NA	NA	0.49 (0.39-0.60)	<.001
Genetics*Telehealth	NA	NA	NA	NA	4.37 (3.17-6.00)	<.001
Mental Health*Telehealth	NA	NA	NA	NA	0.60 (0.53-0.68)	<.001
OBYGN*Telehealth	NA	NA	NA	NA	1.23 (0.83-1.81)	.30
Oncology*Telehealth	NA	NA	NA	NA	1.25 (1.00-1.54)	.05
Pulmonology*Telehealth	NA	NA	NA	NA	0.80 (0.50-1.15)	.20

Abbreviations: NA, not applicable; OR, odds ratio

[a] Reference group is female

[b] Reference group is Established Patient

Supplementary Table4. Stratified Analysis (Hispanic Patients): Adjusted Odds of No-Show Encounters by Encounter Type

	**Hispanic**					
**Characteristics**	Telehealth		In-person		Telehealth and In-person	
**Encounter Type**	OR (95% CI)	P-value	OR (95% CI)	P-value	OR (95% CI)	P-value
In-person	NA	NA	NA	NA	1[Reference]	NA
Telehealth	NA	NA	NA	NA	0.22 (0.18-0.27)	<.001
**Sex**						
Male[a]	0.81 (0.62-1.07)	.13	1.22 (1.15-1.30)	<.001	1.21 (1.13-1.27)	<.001
**Age at first visit**						
18-39	1[Reference]	NA	1[Reference]	NA	1[Reference]	NA
40-64	0.82 (0.62-1.07)	.14	0.83 (0.77-0.87)	<.001	0.83 (0.78-0.88)	<.001
65+	0.84 (0.51-1.37)	.48	0.60 (0.53-0.67)	<.001	0.61 (0.54-0.68)	<.001
**Insurance**						
Commercial	1[Reference]	NA	1[Reference]	NA	1[Reference]	NA
Medicaid	1.44 (1.09-1.90)	.01	2.10 (1.96-2.25)	<.001	2.10 (1.96-2.25)	<.001
Medicare	1.59 (1.10-2.28)	.01	1.53 (1.38-1.71)	<.001	1.53 (1.38-1.70)	<.001
Uninsured	2.22 (1.27-3.88)	.005	1.88 (1.66-2.14)	<.001	1.89 (1.47-2.14)	<.001
**Median Household Income**						
<$50,000	1[Reference]	NA	1[Reference]	NA	1[Reference]	NA
$50,000-100,000	0.88 (0.67-1.17)	.36	0.85 (0.79-0.91)	<.001	0.85 (0.78-0.91)	<.001
$100,000+	0.89 (0.61-1.28)	.52	0.74(0.67-0.82)	<.001	0.74 (0.67-0.82)	<.001
**Type of visit**						
New Patient[b]	1.38 (1.05-1.82)	.02	0.99 (0.94-1.05)	.67	1.00 (0.95-1.05)	.16
**Charlson Comorbidity Index**						
0	1[Reference]	NA	1[Reference]	NA	1[Reference]	NA
1-2	1.14 (0.89-1.47)	.30	1.11 (1.04-1.10)	.003	1.11 (1.04-1.19)	.002
3+	1.01 (0.74-1.37)	.97	1.31 (1.20-1.43)	<.001	1.29 (1.18-1.41)	<.001
**Distance to Healthcare Facility**						
Q1 (0-3.1 miles)	1[Reference]	NA	1[Reference]	NA	1[Reference]	NA
Q2 (3.2-6.0miles)	0.93 (0.64-1.35)	.68	1.01 (0.93-1.11)	.77	1.01 (0.93-0.1.10)	.85
Q3(6.1-12.2 miles)	1.14 (0.81-1.57)	.46	1.16 (1.07-1.26)	.001	1.16 (1.07-1.25)	<.001
Q4 (12.3 miles+)	1.7 (0.76-1.50)	.71	1.07 (0.98-1.17)	.15	1.07 (0.97-1.17)	.14
**Specialty**						
Primary Care	1[Reference]	NA	1[Reference]	NA	1[Reference]	NA
Cardiology	1.51 (0.66-3.49)	.33	1.02 (0.91-1.15)	.70	1.03 (0.91-1.15)	.68
Dermatology	0.90 (0.32-2.50)	.84	1.50 (1.38-1.62)	<.001	1.49 (1.38-1.62)	<.001
Endocrinology	0.50 (0.29-0.86)	.01	1.11 (1.06-1.17)	<.001	1.30 (1.19-1.43)	<.001
Genetics	2.84 (1.79-4.48)	<.001	1.23 (0.53-1.30)	.31	0.80 (0.53-1.21)	.30
Mental Health	3.38 (2.62-4.38)	<.001	2.30 (2.02-2.66)	<.001	2.32 (2.01-2.66)	<.001
OBGYN	2.23 (1.25-3.99)	.007	0.85 (0.79-0.92)	<.001	0.85 (0.78-0.92)	<.001
Oncology	1.73 (1.06-2.82)	.03	0.55 (0.50-0.61)	<.001	0.55 (0.50 -0.61)	<.001
Pulmonology	0.58 (0.14-2.31)	.44	1.27 (1.12-1.45)	<.001	1.27 (1.12-1.45)	<.001

**Time**						
Jan – Mar, 2021	0.78 (0.53-1.15)	.21	0.85 (0.78-0.93)	<.001	0.85 (0.94-1.08)	<.001
Apr – Jun, 2021	1[Reference]	NA	1[Reference]	NA	1[Reference]	NA
Jul – Sep, 2021	1.00 (0.70-1.42)	.99	1.00 (0.93-1.08)	.94	1.00 (0.93-1.08)	.93
Oct – Dec, 2021	0.90 (0.63-1.30)	.59	0.89 (0.82-0.95)	.002	0.89 (0.83-0.96)	.001
Jan – Mar, 2022	1.04 (0.76-1.42)	.82	0.82 (0.76-0.89)	<.001	0.83 (0.77-0.89)	<.001
Apr – Jun, 2022	1.14 (0.97-1.85)	.08	0.99 (0.93-1.08)	.98	1.01 (0.94-1.08)	.80
**Interactions**						
Medicaid*Telehealth	NA	NA	NA	NA	0.69 (0.52-0.91)	.009
Medicare*Telehealth	NA	NA	NA	NA	0.98 (0.73-1.33)	.92
Uninsured*Telehealth	NA	NA	NA	NA	1.17 (0.67-2.03)	.58
Cardiology*Telehealth	NA	NA	NA	NA	1.45 (0.63-3.36)	.39
Dermatology*Telehealth	NA	NA	NA	NA	0.65 (0.23-1.79)	.40
Endocrinology*Telehealth	NA	NA	NA	NA	0.41 (0.24-0.71)	.001
Genetics*Telehealth	NA	NA	NA	NA	4.99 (2.79-8.89)	<.001
Mental Health*Telehealth	NA	NA	NA	NA	1.47 (1.11-1.93)	.006
OBYGN*Telehealth	NA	NA	NA	NA	3.43 (1.94-16.04)	<.001
Oncology*Telehealth	NA	NA	NA	NA	3.14 (1.96-5.02)	<.001
Pulmonology*Telehealth	NA	NA	NA	NA	0.48 (0.12-1.90)	.29

Abbreviations: NA, not applicable; OR, odds ratio

[a] Reference group is female

[b] Reference group is Established Patient

Supplmentary Table5. Stratified Analysis (Insurance): Adjusted Odds of No-Show Encounters

Characteristics	Commerical		Medicare		Medicaid		Uninsured	
**Encounter Type**	OR (95% CI)	P-value	OR (95% CI)	P-value	OR (95% CI)	P-value	OR (95% CI)	P-value
**In-person**	1[Reference]	NA	1[Reference]	NA	1[Reference]	NA	1[Reference]	NA
**Telehealth**	0.38 (0.35-0.42)	<.001	0.40 (0.35-0.46)	<.001	0.15 (0.11-0.18)	<.001	0.30 (0.19-0.46)	<.001
**Sex**								
Male[a]	1.36 (1.31-1.40)	.13	1.06 (1.01-1.12)	.003	1.17 (1.08-1.25)	<.001	1.40 (1.21-1.62)	<.001
**Age at first visit**								
18-39	1[Reference]	NA	1[Reference]	NA	1[Reference]	NA	1[Reference]	NA
40-64	0.83 (0.80-0.86)	<.001	0.73 (0.60-0.88)	.001	0.80 (0.75-0.86)	<.001	0.74 (0.64-0.85)	<.001
65+	0.72 (0.66-0.79)	<.001	0.57 (0.47-0.67)	<.001	0.74 (0.64-0.76)	<.001	0.63 (0.48-0.83)	.001
**Median Household Income**								
<$50,000	1[Reference]	NA	1[Reference]	NA	1[Reference]	NA	1[Reference]	NA
$50,000-100,000	0.80 (0.76-0.84)	<.001	1.00 (0.93-1.08)	.992	0.78 (0.73-0.84)	<.001	0.66 (0.48-0.82)	<.001
$100,000+	0.84 (0.79-0.88)	<.001	0.97 (0.88-1.05)	.353	0.72 (0.79-0.65)	<.001	0.65 (0.52-0.81)	<.001
**Race/Ethnicity**								
White	1[Reference]	NA	1[Reference]	NA	1[Reference]	NA	1[Reference]	NA
Black	2.90 (2.76-3.04)	<.001	3.13 (2.92-3.34)	<.001	2.13 (1.97-2.31)	<.001	2.08 (1.77-2.45)	<.001
Hispanic	1.87 (1.78-1.94)	<.001	2.28 (2.11-2.44)	<.001	1.26 (1.17-1.35)	<.001	3.03 (2.52-3.65)	<.001
**Type of visit**								
New Patient[b]	0.90(0.87-0.93)	.02	1.07 (1.02-1.11)	.67	1.14 (1.09-1.20)	<.001	1.16 (1.04-1.31)	.27
**Charlson Comorbidity Index**								
0	1[Reference]	NA	1[Reference]	NA	1[Reference]	NA	1[Reference]	NA
1-2	1.02 (0.98-1.05)	.30	1.28 (1.18-1.40)	<.001	1.16 (1.08-1.25)	<.001	1.31 (1.13-1.52)	<.001
3+	1.14 (1.09-1.21)	.97	1.71 (1.58-1.86)	<.001	1.24 (1.13-1.34)	<.001	1.71 (1.39-2.10)	<.001
**Distance to Healthcare Facility**								
Q1 (0-3.1 miles)	1[Reference]	NA	1[Reference]	NA	1[Reference]	NA	1[Reference]	NA
Q2 (3.2-6.0miles)	0.91 (0.99-0.55)	.02	0.91 (0.85-0.97)	.003	0.92 (0.84-.1.01)	.07	0.83 (0.70-0.99)	.04
Q3(6.1-12.2 miles)	1.04 (1.00-1.09)	.06	1.13 (1.06-1.20)	<.001	1.01 (0.92-1.11)	.82	0.95 (0.79-1.15)	.61
Q4 (12.3 miles+)	1.29 (1.24-1.35)	<.001	1.09 (1.02-1.16)	.007	1.05 (0.96-1.16)	.30	1.02 (0.85-1.23)	.82
**Specialty**								
Primary Care	1[Reference]	NA	1[Reference]	NA	1[Reference]	NA	1[Reference]	NA
Cardiology	1.10 (1.01-1.19)	.03	1.10 (1.03-1.17)	.003	0.87 (0.78-0.97)	.01	0.81 (0.59-1.11)	.20
Dermatology	1.18 (1.13-1.23)	<.001	1.12 (1.05-1.20)	<.001	1.15 (1.06-1.24)	.001	0.94 (0.77-1.15)	.53
Endocrinology	1.11 (1.05-1.18)	<.001	1.49 (1.39-1.59)	<.001	1.12 (1.02-1.23)	.02	1.05 (0.83-1.33)	.69
Genetics	0.74 (0.59-0.93)	.011	0.90 (0.57-1.43)	.66	0.71 (0.49-1.04)	.08	0.26 (0.063-1.07)	.06
Mental Health	3.02 (2.80-3.25)	<.001	7.31 (6.61-8.08)	<.001	1.32 (1.20-1.45)	<.001	3.21 (2.24-4.26)	<.001
OBGYN	0.83 (0.79-0.87)	<.001	1.41 (1.27-1.57)	<.001	0.82 (0.77-0.88)	<.001	0.87 (0.73-1.05)	.15
Oncology	0.83 (0.60-0.67)	<.001	0.77 (0.72-0.81)	<.001	0.51 (0.46 -1.31)	<.001	0.41 (0.32-0.53)	<.001
Pulmonology	1.39 (1.28-1.51)	<.001	1.42 (1.32-1.52)	<.001	1.17 (1.05-1.31)	.004	1.08 (0.79-1.46)	.63
**Time**								
Jan – Mar, 2021	0.86 (0.82-0.90)	<.001	0.97 (0.92-1.02)	.23	0.94 (0.88-1.00)	.05	0.94 (0.88-1.14)	.54

Apr – Jun, 2021	1[Reference]	NA	1[Reference]	NA	1[Reference]	NA	1[Reference]	NA
Jul – Sep, 2021	1.04 (1.00-1.08)	.08	1.10 (1.04-1.16)	<.001	1.00 (0.94-1.06)	.97	1.04 (0.87-1.25)	.65
Oct – Dec, 2021	0.88 (0.85-0.93)	<.001	1.06 (1.01-1.12)	.032	0.93 (0.87-0.97)	.04	0.92 (0.77-1.10)	.34
Jan – Mar, 2022	0.83 (0.79-0.86)	<.001	1.04 (0.99-1.10)	.14	0.87 (0.82-0.93)	<.001	1.02 (0.85-1.20)	.85
Apr – Jun, 2022	0.93 (0.89-0.96)	<.001	1.07 (1.01-1.12)	.02	1.01 (0.95-1.08)	.71	1.00 (0.83-1.21)	.99
**Interactions**								
Hispanic*Telehealth	0.67 (0.57-0.79)	<.001	0.73 (0.55-0.96)	0.03	0.89 (0.66-1.19)	.41	0.79 (0.44-1.44)	.30
Black*Telehealth	0.58 (0.50-0.68)	<.001	0.57 (0.47-0.70)	<.001	0.75 (0.57-0.97)	.13	0.57 (0.31-1.06)	.31
Cardiology*Telehealth	1.27 (0.88-1.83)	.20	1.19 (0.85-1.67)	.30	1.67 (0.76-3.64)	.20	0 (0-0)	.31
Dermatology*Telehealth	0.69 (0.42-1.12)	.13	1.34 (0.49-3.65)	.57	2.10 (0.98-4.51)	.06	1.38 (2.08-9.29)	/.97
Endocrinology*Telehealth	0.57 (0.46-0.71)	<.001	0.17 (0.10-0.29)	<.001	0.59 (0.32-1.081)	.09	1.19 (0.50-2.82)	.44
Genetics*Telehealth	4.47 (3.34-5.99)	<.001	1.65 (0.59-4.65)	.34	3.00 (1.63-5.50)	<.001	5.5 (0.86-3.54)	.95
Mental Health*Telehealth	0.86 (0.76-0.99)	.029	0.42 (0.35-0.51)	<.001	2.85 (2.21-3.67)	<.001	1.27 (0.70-2.28)	.30
OBYGN*Telehealth	1.73 (1.25-2.46)	.002	0.86 (0.35-2.10)	.74	3.44 (1.94-16.04)	<.001	1.88 (0.45-7.94)	.74
Oncology*Telehealth	1.84 (1.45-2.36)	<.001	1.13 (0.84-1.52)	.42	2.85. (1.67-4.86)	<.001	0.89 (0.12-6.78)	1.03
Pulmonology*Telehealth	0.60 (0.32-1.13)	.12	0.68 (0.42-1.12)	.13	0 (0-0)	<.001	0 (0-0)	.71

Abbreviations: NA, not applicable; OR, odds ratio

[a] Reference group is female

[b] Reference group is Established Patient

Supplmenetary Table 6. Study Sample Characteristics of Mental Health Patients

Characteristic	Black		White		Hispanic	
	In-person (n=10,035)	Telehealth (n=6,222)	In-person (n=69,384)	Telehealth (n=30,628)	In-person (n=7,653)	Telehealth (n=4,518)
**No-show Encounters (%)**	1,936 (19.3)	488 (7.8)	9,403(13.6)	935 (3.1)	1,297 (16.9)	229 (5.1)
**Sex, No. (%)**						
Female	6,139 (61.2)	5,145 (82.7)	42,853 (61.8)	21,044 (68.7)	4,759 (62.2)	3,372 (74.7)
Male	3,896 (38.8)	1,077 (17.3)	26,531 (38.2)	9,584 (31.3)	2,894 (37.8)	1,141 (25.3)
**Age at first visit, No. (%)**						
18-39	5,668 (56.5)	2,361 (37.9)	31,803 (45.8)	13,073 (42.7)	4,561 (59.6)	2,446 (54.1)
40-64	3,544 (35.3)	2,923 (47.0)	26,984 (38.9)	12,066 (39.4)	2,699 (35.3)	1,822 (40.3)
65+	823 (8.2)	938 (15.1)	10,597 (15.3)	5,489 (17.9)	393 (5.1)	250 (5.5)
**Primary Payer, No. (%)**						
Commercial	2,201 (21.9)	2,305 (37.0)	28,112 (40.5)	18,903 (61.7)	3.131 (40.9)	2,490 (55.1)
Medicaid	5,304 (52.9)	1,506 (24.2)	20,150 (29.0)	3,008 (9.8)	3,223 (42.1)	1,093 (24.2)
Medicare	2,416 (24.1)	2,350 (37.8)	19,493 (28.1)	8,208 (26.8)	1,002 (13.1)	837 (18.5)
Uninsured	114 (1.1)	61 (1.0)	1,629 (2.3)	509 (1.7)	297 (3.9)	98 (2.2)
**Median Household Income, No. (%)**						
<$50,000	9,018 (63.5)	2,782 (44.7)	17,076 (24.6)	2,384 (7.8)	2,358 (30.8)	1,005 (22.2)
$50,000 – 100,000	4,307 (30.3)	2,566 (41.2)	37,360 (53.8)	17,517 (57.2)	4,381 (57.2)	2,774 (61.4)
$100,000+	884 (6.2)	874 (14.0)	14,948 (21.5)	10,727 (35.0)	914 (11.9)	739 (16.4)
**Type of Visit, No. (%)**						
New Patient	1,660 (11.7)	672 (10.8)	7,923 (11.4)	4,989 (12.6)	1,104 (14.4)	521 (11.5)
Established Patient	8,375 (83.5)	5,550 (89.2)	61,461 (88.6)	25,639 (83.7)	6,549 (85.6)	3,992 (88.5)
**Charlson Comorbidity Index, No. (%)**						
0	6,429 (45.2)	2,048 (32.9)	32,872 (47.4)	13,764 (44.9)	4,561 (56.9)	1,877 (41.5)
1-2	4,648 (32.7)	2,038 (32.8)	22,456 (32.4)	9,658 (31.5)	2,138 (27.9)	1,552 (34.4)
3+	3,132 (22.0)	2,136 (34.3)	14,056 (20.3)	7,206 (23.5)	1,159 (15.1)	1,089 (24.1)
**Distance to Healthcare Facility, No. (%)**						
Q1 (0-3.1 miles)	2,743 (19.3)	1,090 (17.5)	9,272 (13.4)	7,483 (24.4)	738 (9.6)	597 (13.2)
Q2 (3.2-6.0 miles)	6,269 (44.1)	939 (15.1)	28.692 (41.4)	7,610 (24.8)	3,045 (39.8)	1,002 (22.2)
Q3 (6.1-12.2 miles)	3,102 (21.8)	2,516 (40.4)	16,927 (24.4)	7,316 (23.9)	1,900 (24.8)	1,743 (38.6)
Q4 (12.3 miles+)	2,095 (14.7)	1,677(27.0)	14,493 (20.9)	8,219 (26.8)	1,970 (25.7)	1,176 (26.0)
**Time, No. (%)**						
Jan – March, 2021	1,461 (14.6)	598 (9.6)	9,371 (13.5)	3,305 (10.8)	885 (11.6)	407 (9.0)
Apr - Jun, 2021	1,348 (13.4)	1,062(17.1)	9,492 (13.7)	4,703 (15.4)	898 (11.7)	726 (16.1)
Jul – Sep, 2021	1,447 (14.4)	1,188 (19.1)	10,717 (15.4)	4,949 (16.2)	1,138 (14.9)	758 (16.8)
Oct – Dec, 2021	1,689 (16.8)	1,070 (17.2)	12,914 (18.6)	5,130 (16.7)	1,450 (18.9)	774 (17.1)
Jan – Mar, 2022	1,972 (19.3)	1,298 (20.9)	13,134 (18.9)	6,596 (21.5)	1,616 (21.1)	1,006 (22.3)
Apr – Jun, 2022	2,118 (21.1)	1,006 (16.2)	13,756 (19.8)	5,945 (19.4)	1,666 (21.8)	847 (18.7)

Supplementary Figure 1. Temporal Trends in No-Show Rates Among Mental Health Patients

a.

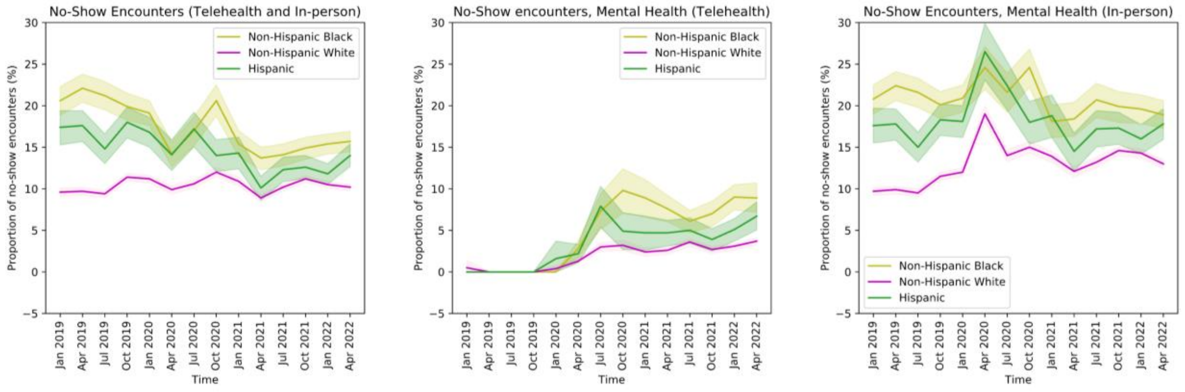

b.

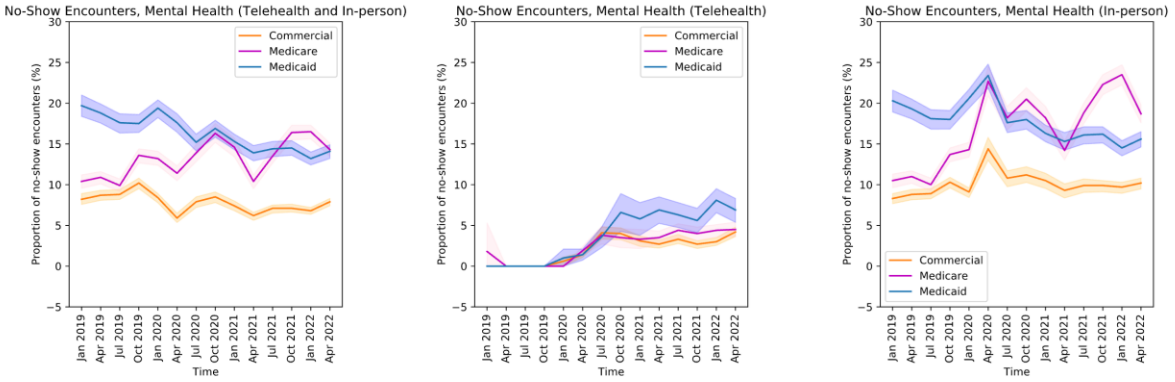

Supplementary Table 7. Adjusted Odds of No-Show Encounters Among Mental Health Patients by Encounter Type

	Telehealth only		In-person only		Telehealth and in-person	
**Characteristics**	OR (95% CI)	P-value	OR (95% CI)	P-value	OR (95% CI)	P-value
**Encounter type**						
In-person	NA	NA	NA	NA	1[Reference]	NA
Telehealth	NA	NA	NA	NA	0.27 (0.25-0.29)	<.001
**Race/ethnicity**						
White	1[Reference]	NA	1[Reference]	NA	1[Reference]	NA
Black	2.13 (1.79-2.55)	.001	1.46 (1.28-1.67)	<.001	1.65 (1.46-1.87)	<.001
Hispanic	1.41 (1.15-1.73)	<.001	1.34 (1.18-1.53)	<.001	1.41 (1.25-1.60)	<.001
**Sex**						
Male[a]	1.10 (0.95-1.28)	.212	1.25 (1.15-1.36)	<.001	1.23 (1.14-1.33)	.088
**Age at first visit**						
18-39	1[Reference]	NA	1[Reference]	NA	1[Reference]	NA
40-64	0.87 (0.74-1.01)	.07	0.88 (0.79-0.98)	.01	0.88 (0.80-0.97)	.010
65+	0.51 (0.39-0.67)	<.001	2.17 (1.85-2.55)	<.001	1.82 (1.57-2.10)	<.001
**Payer**						
Commercial	1[Reference]	NA	1[Reference]	NA	1[Reference]	NA
Medicaid	1.70 (1.41-2.05)	<.001	1.81 (1.62-2.01)	<.001	1.74 (1.57-1.92)	<.001
Medicare	1.43 (1.17-1.76)	.001	1.24 (1.07-1.43)	.04	1.26 (1.11-1.44)	<.001
Uninsured	2.13 (1.44-3.16)	<.001	1.84 (1.41-2.39)	<.001	1.84 (1.45-2.34)	<.001
**Median Household Income**						
<$50,000	1[Reference]	NA	1[Reference]	NA	1[Reference]	NA
$50,000-100,000	0.78 (0.65-0.95)	.01	1.01 (0.90-1.14)	.87	0.99 (0.89-1.11)	.92
>$100,000	0.67 (0.53-0.85)	.001	1.09 (0.94-1.26)	.26	1.06 (0.93-1.21)	.37
**Type of visit**						
New Patient[b]	1.12 (0.96-1.31)	.15	1.19 (1.12-1.26)	<.001	1.21 (1.14-1.28)	<.001
**Charlson Comorbidity Index**						
0	1[Reference]	NA	1[Reference]	NA	1[Reference]	NA
1-2	1.06 (0.91-1.24)	.46	1.28 (1.15-1.43)	<.001	1.27 (1.15-1.39)	<.001
3+	1.15 (0.95-1.39)	.13	1.74 (1.53-1.97)	<.001	1.65 (1.48-1.85)	<.001
**Distance to healthcare facility**						
Q1 (0-3.1 miles)	1[Reference]	NA	1[Reference]	NA	1[Reference]	NA
Q2 (3.2-6.0 miles)	0.84 (0.67-1.06)	.14	0.95 (0.83-1.09)	.44	0.94 (0.83-1.06)	.32
Q3 (6.1-12.2 miles)	0.90 (0.73-1.11)	.33	1.29 (1.12-1.49)	<0.001	1.31 (1.15-1.48)	<.001
Q4 (12.3 miles+)	0.98 (0.80-1.19)	.81	1.54 (1.34-1.77)	<0.001	1.51 (1.34-1.71)	<.001
**Time**						

January – March, 2021	1.00 (0.79-1.27)	.98	1.19 (1.06-1.33)	.003	1.17 (1.05-1.30)	.003
April – June, 2021	1[Reference]	NA	1[Reference]	NA	1[Reference]	NA
July – September, 2021	1.15 (0.96-1.39)	.14	1.10 (0.99-1.23)	.07	1.11 (1.01-1.22)	.03
October – December, 2021	0.96 (0.79-1.17)	.70	1.15 (1.03-1.28)	.01	1.13 (1.02-1.25)	.02
January – March, 2022	1.17 (0.98-1.41)	.08	1.12 (1.01-1.26)	.04	1.13 (1.03-1.25)	.01
April – June, 2022	1.37 (1.14-1.64)	.001	1.04 (0.93-1.17)	.46	1.08 (0.98-1.20)	.11
Interactions						
Black*Telehealth	NA	NA	NA	NA	1.65 (1.33-2.04)	<.001
Hispanic*Telehealth	NA	NA	NA	NA	1.16 (0.90-1.49)	.24
Medicaid*Telehealth	NA	NA	NA	NA	0.95 (0.77-1.19)	.67
Medicare*Telehealth	NA	NA	NA	NA	0.50 (0.41-0.61)	<.001
Uninsured*Telehealth	NA	NA	NA	NA	1.09 (0.66-1.80)	.35

Abbreviations: NA, not applicable; OR, odds ratio

[a] Reference group is female

[b] Reference group is Established Patient

Supplementary Information 1

Figure 2b presents no-show rates for in-person and telehealth encounters stratified by different types of insurance. In the last quarter of 2019, no-show rates notably varied by insurance type (15.0% [95%CI, 14.51% -15.50%] Medicaid, 9.7% [95%CI, 8.80% - 10.50%] Uninsured, 5.3% [95%CI, 5.20%-5.40%] Commercial, 5.0% [95%CI, 4.83%-5.00%] Medicare). Similar to the trends described earlier, the overall no-show rate significantly decreased relative to pre-pandemic times with the increased use of telehealth (12.5% [95%CI, 12.11% -12.80%] Medicaid, 6.6% [95%CI, 6.01% - 7.10%] Uninsured, 3.5% [95%CI, 3.41%-3.50%] Commercial, 3.8% [95%CI, 3.69%-3.90%] Medicare). Within telehealth encounters, the difference in no-show rates among patients with different insurance groups are notably lower (4.8% [95%CI, 3.43% -4.80%] Medicaid, 2.5 % [95%CI, 1.31%-3.70%] Uninsured, 1.9 % [95%CI, 1.69%-2.00%] Commercial, 1.8% [95%CI, 1.55%-2.00%] Medicare).

Supplementary Information 2

For mental health specialty, we ran analyses focused just on 22,320 patients seen in mental health specialty comprised of 173,282 total encounters (53,373 [30.80%] telehealth, 119,909 [69.2%] in-person) as shown in **eTable 6**. We demonstrated that the trends in usage and disparities in telehealth are consistent with other analyses that were aggregated across specialties (**eFigure 1** and **eFigure 2**). **eTable 7** shows the ORs of having a no-show encounter from the multivariate GEE-adjusted logistic regression analysis for the mental health care exhibits similar trends as the overall trend. No-show odds are reduced when using telehealth (OR, 0.27; 95% CI, 0.25-0.29). In contrast to the overall trend, patients in mental health care who are 65 and older had significantly higher no-show odds for in-person encounters (OR, 2.17; 95% CI, 1.85-2.55). Using telehealth, the no-show odds for 65 and older are reduced (OR, 0.51; 95% CI, 0.39-0.67) relative to the reference age group. Interestingly, Black patients receiving mental health care appeared to have high no-show odds for telehealth (OR, 2.13; 95% CI, 1.79-2.55) and moderately high no-show odds for in-person (OR, 1.46; 1.28-1.67) relative to White patients receiving mental health care. The opposite trend was true for the no-show odds with all specialties (OR, 1.29 [95% CI, 1.14-1.46] Black telehealth, OR, 2.85 [95% CI, 2.74-2.96] Black in-person).

Supplementary Information 3

In situations that are desirable to increase telehealth usage among specialties with low overall usage, aside from payment parities at the federal and private levels[42], it will be critical to select an easy and accessible telehealth infrastructure and invest in telehealth education for physicians to dismantle any concerns about quality, privacy, data security, and financial/legal regulations that may be obstructing implementation and efficient clinical workflow.[43,44] As physicians serve

as initial gatekeepers to the implementation and usage of telehealth, it is imperative to keep physicians' needs that may be unique to each specialty to successfully implement a sustainable healthcare system and ensure they have trust in the system.[45,46] Of course, on the flip side, it is important to ensure that patients feel like they can also trust the quality of care they receive from telehealth visits and ensure that their privacy is protected. This is critical for specialties like OBGYN where patients may more likely feel vulnerable and need better trust in the system. For the Sustained group, we should focus on reducing no-show encounters and consider implementing effective reminders through patient's preferred communication method (e.g., text, phone call, email), installing a more dynamic and flexible scheduling policy, and providing patient education.[47-49]

Supplementary Information 4

Of note, there will be fiscal barriers to permanently expand telehealth. Telehealth encounters cannot completely replace in-person encounters for all specialties, but it can substitute for most patient follow-ups and consultations for certain specialties. In theory, telehealth is cheaper. Although if it's utilized excessively for its affordability and ease of access, it may lead to more overall encounters (and no-show encounters) and higher costs for both public and private insurance.[50] Differential visit reimbursement for different type of visits (in-person, video, and telephone) may also lead to perverse incentives to unnecessarily have video-consultations when a telephone call may have been sufficient.[51]

Supplementary Information 5

Supplementary Figure 1. Temporal Trends in No-Show Rates Among Mental Health Patients by Race/Ethnicity.

No-show rate, or percentage of no-show encounters, is determined by dividing the number of no-show encounters by the total number of scheduled encounters, where the total includes no-show, completed, and canceled encounters. Only mental health encounters are included in this temporal analysis. The 95% confidence interval bands represent the range within which we are 95% confident that the values for the observed trend data points lie. **a** Difference racial and ethnic groups exhibited varying no-show rates for in-person and telehealth appointments, yet telehealth seems to reduce these disparities for mental health specialty **b** No-show rates for in-person and telehealth appointments by insurance type, with Medicaid patients having the highest rates, though these differences narrow with telehealth. Uninsured group is not shown in this graph, as the sample size was too small.
